# Parkinson’s Protein α-Synuclein Binds Efficiently and with a Novel Conformation to Two Natural Membrane Mimics

**DOI:** 10.1371/journal.pone.0142795

**Published:** 2015-11-20

**Authors:** Pravin Kumar, Ine M. J. Segers-Nolten, Nathalie Schilderink, Vinod Subramaniam, Martina Huber

**Affiliations:** 1 Department of Physics, Huygens-Kammerlingh-Onnes Laboratory, Leiden University, Leiden, The Netherlands; 2 Nanobiophysics, MESA+ Institute for Nanotechnology, University of Twente, Enschede, The Netherlands; 3 FOM Institute AMOLF, Amsterdam, The Netherlands; Martin-Luther-Universität Halle-Wittenberg, GERMANY

## Abstract

Binding of human α-Synuclein, a protein associated with Parkinson’s disease, to natural membranes is thought to be crucial in relation to its pathological and physiological function. Here the binding of αS to small unilamellar vesicles mimicking the inner mitochondrial and the neuronal plasma membrane is studied in situ by continuous wave and pulsed electron paramagnetic resonance. Local binding information of αS spin labeled by MTSL at positions 56 and 69 respectively shows that also helix 2 (residues 50–100) binds firmly to both membranes. By double electron-electron resonance (DEER) on the mutant spin labeled at positions 27 and 56 (αS 27/56) a new conformation on the membrane is found with a distance of 3.6 nm/ 3.7 nm between residues 27 and 56. In view of the low negative charge density of these membranes, the strong interaction is surprising, emphasizing that function and pathology of αS could involve synaptic vesicles and mitochondria.

## Introduction

Parkinson’s disease [1] is the second most prevalent neurodegenerative disorder [2], characterized by the formation of intra-neuronal protein deposits such as Lewy bodies [3] [4]. The protein α-synuclein (αS) is the main component of these protein deposits [5] [6]. The protein αS consists of 140 amino acids and lacks a defined secondary structure in solution [7] [8]. Its physiological function is still not clear, although it has been implicated in neurotransmitter release [9] [10] and vesicle trafficking [11]. Both these functions involve the neuronal plasma membrane (NPM). The protein αS is also associated with diseases like dementia, and mitochondrial dysfunction [12] [13] and with aging [14]. In the brain, αS is present in high concentrations in presynaptic nerve terminals, it has been found to be associated with synaptic vesicles [15], and also in glia. The protein also occurs in mitochondria, especially close to the inner mitochondrial membrane[16][17] and it is thought to be associated with mitochondrial damage [18][19].

When αS binds to membranes, it attains an amphipathic α-helical structure from residues 1–100 [20] [21] [22]. The membrane-bound α-helical αS forms either a continuous helix (residues 1–100), referred to as the extended helix, or the horseshoe conformation, sometimes also referred to as the broken helix. The horseshoe conformation consists of a helix 1 (residues 3–37), a turn, and a helix 2 (residues 45–92)[23] [24], recently a different kink position was suggested [25]. Whether αS binds in the horseshoe or the extended conformation to membranes is still controversial, with some reports supporting the horseshoe conformation [26] [27] and others the extended conformation [28] [29]. Langen and coworkers reported that subtle changes in lipid composition or membrane structure have strong effects on the conformation of αS on the membrane [28]. Previously, we found that the extended as well as the horseshoe conformation coexist on LUVs composed of the negatively charged lipid 1-palmitoyl-2-oleoyl-sn-glycero-3-phospho-(1′-rac-glycerol) (POPG) [30]. Here we show that the same is true for SUVs.

The affinity of αS to membranes depends on the negative charge density (ρ) of the membrane, where ρ represents the molar fraction of anionic lipids present in the membrane [31][32][33]. At higher charge density, both αS helices are tightly bound, but at lower charge density, helix 2 dissociates from the membrane [34].

Since not much is known about the detailed interaction of αS with natural membranes, we investigated the interaction of αS with membranes containing lipids that mimic natural membranes. We focus on two membranes [35]: a. the inner mitochondrial membrane (IMM) and b. the neuronal plasma membrane (NPM), presented in the form of small unilamellar vesicles (SUVs). We applied electron paramagnetic resonance (EPR) and investigated the binding of spin- labeled αS making use of the mobility of the spin label as an indicator for local binding. We focus on two positions, 56 and 69 (αS56, αS69), in the helix 2. We also monitor the conformation of αS on these membranes to determine whether αS is in the horseshoe or the extended conformation. For these experiments, αS was spin labeled at two positions, 27 and 56 (αS27/56), and distances between the spin labels were obtained by DEER (Double Electron-Electron Resonance) [36]. The label positions 27 and 56 were chosen because for these labels both horseshoe and extended conformation yield distances that are measurable by DEER [30].

We show that according to EPR, αS binds equally well to the two natural membranes IMM and NPM. In spite of the low negative charge density of the IMM and NPM membranes, helix 2 of αS binds more strongly to these natural membranes than to POPG/POPC model membranes at comparable charge densities. The binding mode differs from what had been observed on model SUVs before. The extended conformation predominates and the second fraction is a horseshoe with a larger opening angle than previously found.

## Materials and Methods

### Protein expression and labelling

Mutagenesis, protein expression and purification were performed as described previously [37] [38]. Spin labelling was also done following the standard protocol. Briefly, before starting labelling, αS cysteine mutants were reduced with a six-fold molar excess per cysteine with DTT (1,4-dithio-D-threitol) for 30 min at room temperature. To remove DTT, samples were passed twice through Pierce Zeba 5 ml desalting columns. Immediately, a ten-fold molar excess of the MTSL spin label [(1-oxyl-2,2,5,5-tetramethylpyrroline-3-methyl))-methanethiosulfonate] was added (from a 25 mM stock in DMSO) and incubated for 1 h in the dark at room temperature. After this, free spin label was removed by using two additional desalting steps. Protein samples were applied onto Microcon YM-100 spin columns to remove any precipitated and/or oligomerised proteins and diluted in buffer (10 mM Tris-HCl, pH 7.4). Spin label concentrations for single-cysteine mutants were 2.5 mM and for double-cysteine mutants 5 mM at protein concentrations of 250 μM. Owing to the high reactivity of the label and the fact that the cysteine residues are freely accessible in the intrinsically disordered structure, near quantitative labelling can be achieved under these conditions [22]. Samples were stored at -80°C.

### Preparation of vesicles

The lipid compositions for making SUVs were:

- IMM = 1',3'-bis[1,2-dioleoyl-*sn*-glycero-3-phospho]-*sn*-glycerol (CL): 1-palmitoyl-2-oleoyl-*sn*-glycero-3-phosphoethanolamine (POPE): 1-palmitoyl-2-oleoyl-*sn*-glycero-3-phosphocholine (POPC):: 4: 3: 5 [16]
- NPM = L-α-phosphatidylserine (Brain, Porcine) (brain PS): L-α-phosphatidylethanolamine (Brain, Porcine) (brain PE): cholesterol (ovine wool) (CH):: 2: 5: 3 [39]
- POPG SUV's as reference = 100% 1-palmitoyl-2-oleoyl-sn-glycero-3-phospho-(1′-rac-glycerol) (POPG)

All lipids were purchased from Avanti Polar Lipids, Inc. as chloroform solutions and were used without further purification. Lipids were mixed in the desired ratio and then chloroform was evaporated by dry nitrogen gas. The resulting lipid films were kept under vacuum overnight. Dried lipid films were hydrated with 10 mM Tris-HCl, pH 7.4 for 1 hour at 30°C, and the resulting milky lipid suspensions were sonicated for approximately 30 min to make SUVs. The size of the vesicles was determined by dynamic light scattering (DLS). The DLS-experiments were performed on a Zetasizer Nano-ZS (Malvern). We obtained vesicles with a homogeneous size distribution around d = 35 nm (NPM) and 40 nm (IMM and POPG SUVs).

### Sample Preparation

Aliquots of αS from stock solutions (concentration between 150 μM and 250 μM) were added to the SUVs to obtain a lipid to protein ratio (L: P) of 250: 1, and incubated for 30 min at room temperature before measuring. All samples were prepared and measured at least three times. Frozen samples for continuous wave (cw) low-temperature EPR measurements and distance measurements were prepared using 25% spin-labeled and 75% wild type (unlabeled) αS (diamagnetic dilution). The diamagnetically diluted protein mixtures were mixed with the SUVs at a L: P ratio of 250: 1 and incubated for 30 min at room temperature. Glycerol (20% (v/v)) was added to all samples before transferring them into the 3 mm (outer diameter) quartz tubes. The sample tubes were plunged into liquid nitrogen for fast freezing.

### Continuous wave-EPR experiments

The X-band continuous wave (cw) EPR measurements have been performed using a. an EMX PLUS EPR spectrometer (Bruker, Rheinstetten, Germany) with a super high Q cavity (ER 4119 HS-W1) for room temperature measurements and b. an ELEXSYS E680 spectrometer (Bruker, Rheinstetten, Germany) with a rectangular cavity (ER 4102 ST) for low temperature measurements. The room temperature measurements were done at 20°C, using 0.6315 mW of microwave power, 100 kHz modulation frequency and a modulation amplitude of 1.0 G. Total time to acquire EPR spectra was 20 min. The low-temperature measurements were done at 120 K using a helium gas-flow cryostat (Oxford Instruments, United Kingdom) with an ITC502 temperature controller (Oxford Instruments). The EPR spectra were acquired using a modulation amplitude of 2.5 G and a microwave power of 0.6315 mW.

### Simulation of cw-EPR spectra

Spectral simulation was performed using Matlab (7.11.0.584, Natick, Massachusetts, U.S.A) and the EasySpin package [40]. For all simulations, the following spectral parameters were used: g = [2.00906, 2.00687, 2.00300][41] and the hyperfine tensor parameters A_XX_ = A_YY_ = 13 MHz. Usually a superposition of more than one component was required to simulate the spectra. The parameters were manually changed to check in which range acceptable simulations of the experimental spectra were obtained to determine the error margins. The rotation correlation time (*τ*_*r*_) of spin-labelled αS in solution, i.e. in the absence of the membrane was shown to have an error of ± 0.02 ns. To simulate spectra of αS bound to membranes, the *τ*_*r*_ of the fastest component was kept at the *τ*_*r*_ value of the solution spectra of the respective mutant.

### DEER experiments

All DEER experiments were done at X-band on an ELEXSYS E680 spectrometer (Bruker, Rheinstetten, Germany) using a 3 mm split-ring resonator (ER 4118XMS-3-W1). We performed the measurements at 40 K with a helium gas flow using a CF935 cryostat (Oxford Instruments, United Kingdom). The pump and observer frequencies were separated by 70 MHz and adjusted as reported before [26]. The pump-pulse power was adjusted to invert the echo maximally [42]. The pump- pulse length was set to 16 ns. The pulse lengths of the observer channel were 16 and 32 ns for π/2- and π - pulses, respectively. A phase cycle (+ x) -(- x) was applied to the first observer pulse. The complete pulse sequence is given by: ${\frac{\pi }{2}}_{\text{obs}}-\tau_{1}-\pi_{\text{obs}}-\text{t}-\pi_{\text{pump}}-(\tau_{1}+\tau_{2}-\text{t})-\pi_{\text{obs}}-\tau_{2}-\text{echo}.$ The DEER time traces for ten different τ_1_ values spaced by 8 ns starting at τ_1_ = 200 ns were added to suppress proton modulations. Typical accumulation times per sample were 16 hours.

### DEER Analysis

In order to analyze the DEER traces and extract the distance distributions, the software package “DeerAnalysis 2011” was used [43]. Experimental background functions were derived from DEER traces of membrane-bound singly labeled αS under conditions of diamagnetic dilution. The distance distribution was derived by the model free Tikhonov regularization [42] [43]. The distance distributions obtained from the Tikhonov regularization were then fitted using two Gaussians. Errors in the amount by which each fraction contributes to the two distances were determined by changing the parameter “amplitude” of the two Gaussian separately to determine the range which results in an acceptable fit.

## Results

To be sure of the integrity of the vesicles, all SUVs were checked by DLS before and after adding αS. The vesicles were found to have a diameter d = 40 nm for IMM and POPG SUVs and d = 35 nm for NPM, values that did not change upon adding αS.

### Continuous-wave EPR of αS

**Fig 1:**
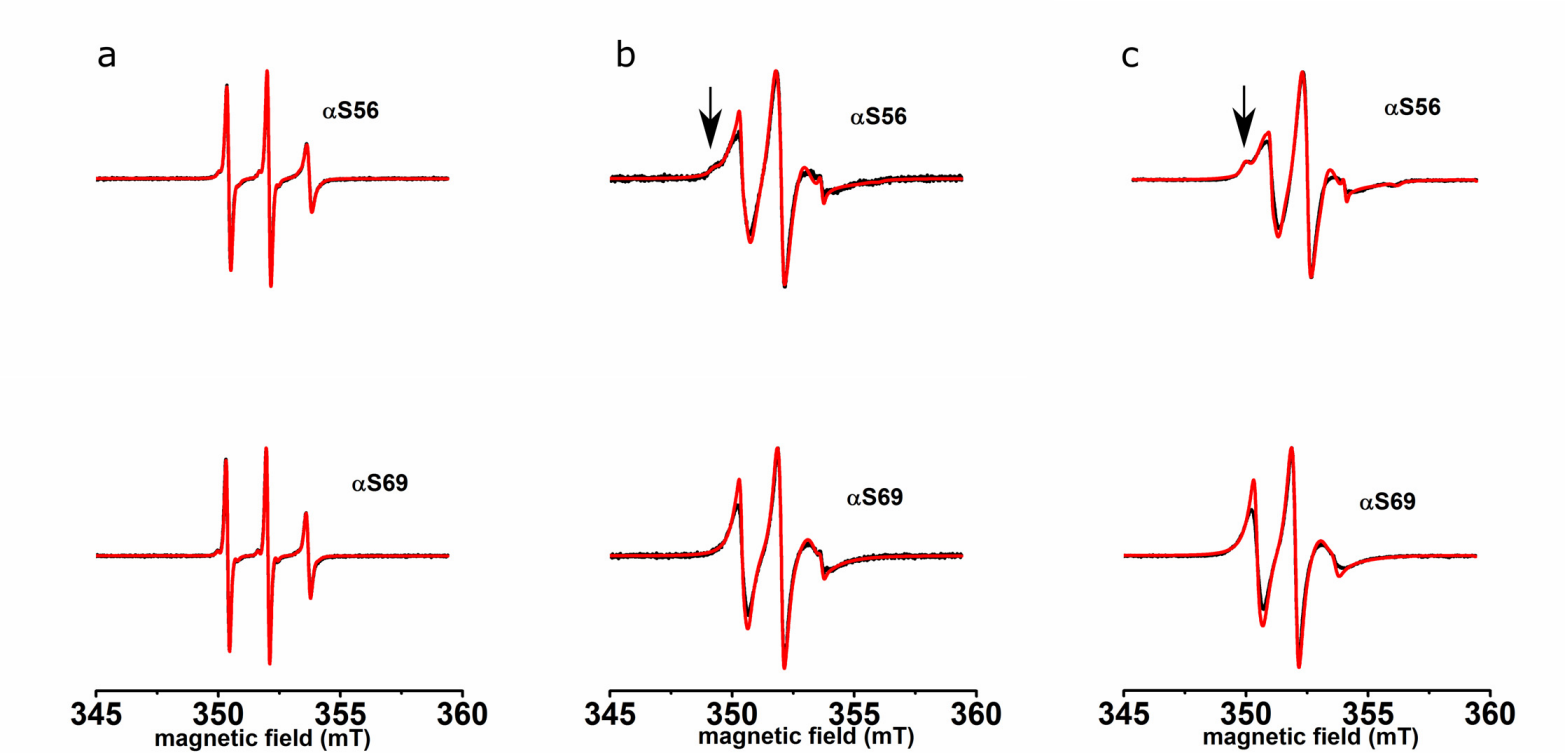
Room temperature, solution EPR spectra of αS56 and αS69 (a) in buffer, (b) with IMM, (c) with NPM. Black line: experiment, red line: simulation. Arrows show the low field feature indicating reduced mobility (see text).

Fig 1A shows the spectra of αS56 and αS69 in buffer solution, measured at room temperature. The spectra of αS56 and αS69 both consist of three narrow lines. Fig 1B and 1C show the spectra of αS in the presence of IMM and NPM respectively. For both αS56 and αS69, the spectral lines are broadened relative to those in Fig 1A. The EPR spectrum of αS56 shows an additional feature, indicated by the arrow in Fig 1.

More detailed information was obtained by spectral simulation of the experimental spectra, which yields the parameters of mobility of the spin label, the rotation correlation time *τ*_*r*_ and, in the case of multicomponent spectra, the amount by which each fraction contributes. These parameters are given in Table 1. The solution spectra are simulated with a single component. The *τ*_*r*_ of αS56 is longer than that of αS69. The spectra of αS56 bound to the IMM and NPM membranes consist of a superposition of three and those of αS69 of two components. The *τ*_*r*_ of the fast component in all spectra was fixed to the *τ*_*r*_ of the respective mutants in solution. The contribution of this fraction to the total spectrum is smaller than 2.5% for each spectrum. The slow components with *τ*_*r*_ values between 2 and 3 ns, contribute at least 87% and αS56 additionally has an immobile component in the order of 10%. The *τ*_*r*_ values and contributions for each mutant are the same within the error margin for IMM and NPM.

**Table 1:** Parameters (τ_r_) describing the mobility of the spin label of αS bound to natural membranes from simulations of cw-EPR spectra. τ_r_: rotation-correlation time.

condition	αS spin label positions	fast component		slow component		immobile component	
		*τ*_*r*_ (ns)	contribution (%)	*τ*_*r*_ (ns)	contribution (%)	*τ*_*r*_ (ns)	contribution (%)
** buffer**	αS56	0.45± 0.02	100	na	na	na	na
	αS69	0.31±0.02	100	na	na	na	na
**IMM**	αS56	0.45	2.0±0.5	2.88±0.13	90 ±1.5	>50.0	8.0
	αS69	0.31	2.0±0.5	2.23±0.11	98 ±1.0	na	na
**NPM**	αS56	0.45	2.0±0.5	2.95±0.14	88±1.5	>50.0	10.0
	αS69	0.31	2.2±0.3	1.99±0.13	98±0.3	na	na

na: not contributing in the simulation. For error determination, see Materials and Methods.

For comparison, the *τ*_*r*_ values for the mutant αS69 on POPG SUVs (34) are 0.39 ± 0.02 ns (for the fast component) and 2.9 ± 0.3 ns (for the slow component), which is longer than found for the IMM and NPM membranes here. We attribute the reduced motion of the nitroxides on POPG vesicles to stronger binding because of the higher negative charge density of POPG SUVs and other factors, such as differences in head-group structure of lipids.

### Results of DEER experiments

**Fig 2:**
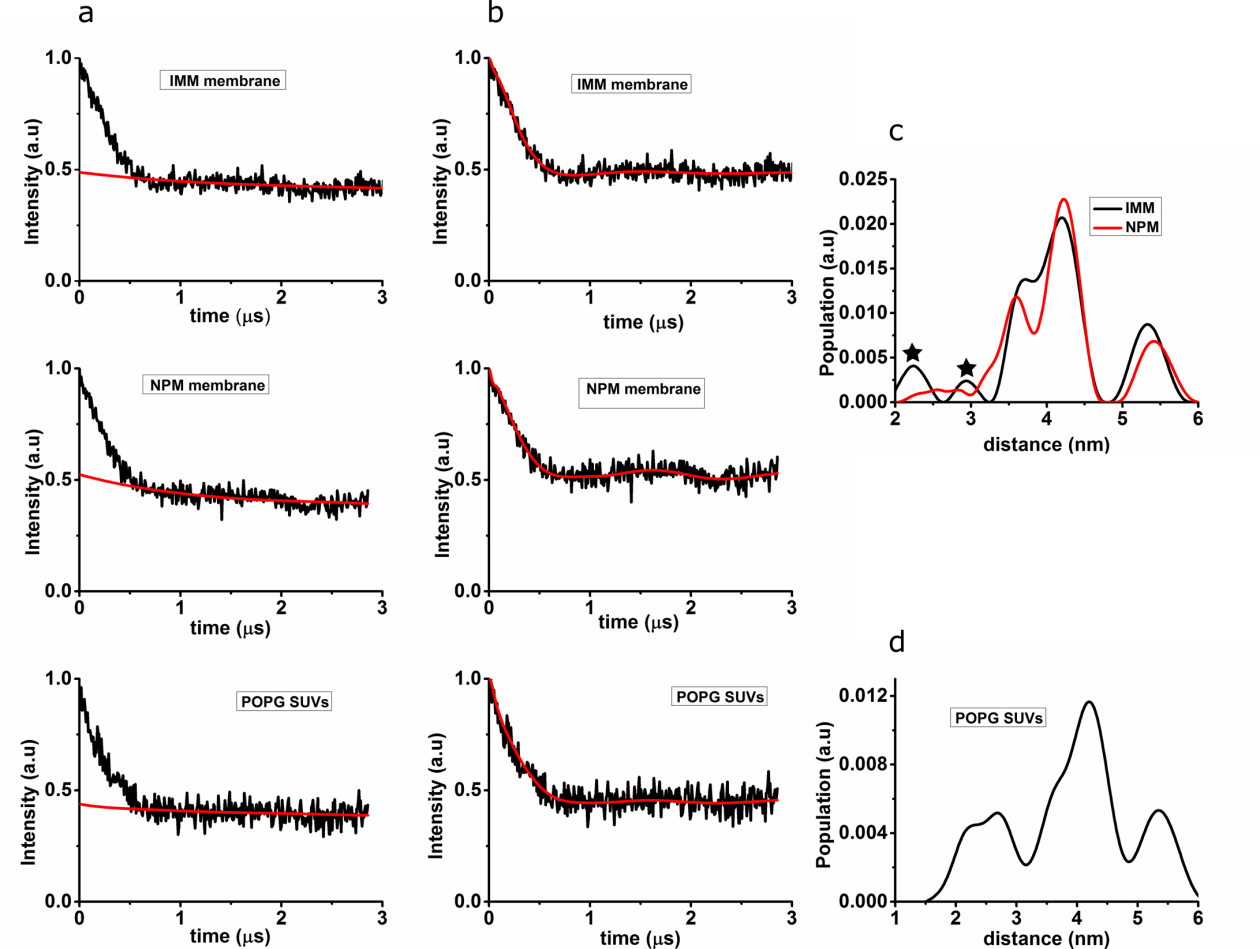
DEER time traces and distance distributions for αS27/56 bound to IMM, NPM and POPG SUVs. (a) Time trace before background correction (black line), red line: background. (b) Time trace after background correction (black line), red line: fit of the time trace with the distance distributions shown in c. (c) Distance distribution obtained after Tikhonov regularization of αS27/56 bound to IMM (black line) and NPM (red line). (d) Distance distribution obtained after Tikhonov regularization of αS27/56 bound to POPG SUVs. For comparison, the same regularization parameter was used for c and d, it seems to be on the small side for d. Small peaks shown with asterisks in Fig 2C have negligible contribution to the distance distribution according to the suppression tool in DEER analysis, the feature at 5.5. nm in all distributions is related to a background artefact and not relevant. Considering the two contributions in the distance distributions, the larger intensity contribution is centered at a longer distance and the smaller intensity contribution is centered at a shorter distance. The distance distributions were fitted with two Gaussians, the parameters of which are given in Table 2.

Fig 2 shows the DEER results obtained for αS27/56 bound to IMM, NPM and POPG SUVs; in Fig 2A the raw experimental DEER time traces before the background correction are displayed, in Fig 2B the experimental time traces after background correction.

The DEER time traces were analyzed by Tikhonov regularization and the resulting distance distributions are shown in Fig 2C (for IMM and NPM) and Fig 2D (for POPG SUVs). The DEER traces show modulation, i.e. a periodic oscillation of the echo intensity as a function of the time *t*, see for example the maximum around 1.7 μs (Fig 2A for αS on NPM membranes). The oscillation is the Fourier Transform of the frequency of the dipolar coupling between the unpaired electron spins of the two nitroxides. The dipolar coupling reflects the distances between the spins in the ensemble. The shape of the time traces obtained in the experiments is analyzed in terms of the distance distributions. The optimized distance distributions (Fig 2C and 2D) result in the fits shown as red lines in Fig 2B. Different methods of analysis were tried for αS on IMM and NPM, revealing that the experimental data is not well reproduced with a single, Gaussian distance distribution, showing that the data cannot be explained by a single, broad distribution of distances, as would be expected for a continuous spread in conformations.

## Discussion

In this study, we investigate the binding of αS to natural membranes by spin-label EPR. The membrane is presented in the form of SUVs, composed of lipids that mimic the natural membranes IMM and NPM. To check the binding of helix 2 of αS to these membranes, cw-EPR at room temperature was performed with spin-label positions representative of helix 2 binding, positions 56 and 69. All spectra reveal clear changes in lineshape in the presence of IMM and NPM showing that αS interacts with these membranes. The spectra and the spectral lineshape simulation parameters (given in Table 1) of αS on IMM and NPM agree within experimental uncertainty, showing that the interaction of αS with both membranes is similar. The fast fraction of αS spin labelled at position 56 and 69 is below 2.5% (Table 1), which shows that helix 2 is firmly bound to the membrane.

When considering only the amount of negative charge density (ρ) of the membranes investigated, IMM (ρ = 0.3) and NPM (ρ = 0.2), the tight binding of helix 2 is surprising, since on model membranes studied previously, αS binding is strongest with vesicles composed exclusively of anionic phospholipids (ρ = 1) [20][33][44]. On SUVs of mixtures of zwitterionic and anionic lipids with a charge density of ρ = 0.26, which is comparable to IMM and NPM membranes, the local degree of binding of αS, when monitored at spin label position 69 of helix 2 is even so low that the bound fraction is too small to be reliably detected by EPR [34]. Therefore, other factors than membrane charge must be responsible for the binding behavior of αS. Several studies have shown such effects [44][45][46][47]. In the present case, the specific lipid composition, for example the CL content must play a role, as already shown by Zigoneanu et al.[45] and Robotta et al.[46]. The lipid CL has a very small head group area compared to the head group of other synthetic lipids such as POPC and POPG, along with a tail region, which consists of four acyl chains. Why this inverted-cone-shaped lipid promotes αS binding is presently unclear, however, it is a likely candidate to promote binding of αS on our IMM SUVs as well. This could be tested by measurements on membranes with different amounts of CL as done in ref. [45] and [46]. The second natural membrane we investigated, NPM, does not contain CL, so it is not clear what causes binding comparable to that to IMM mimics. Besides membrane charge and CL content, several other factors, for example, membrane phase, lipid saturation [27] and posttranslational modification of αS [47] were shown to influence the affinity of αS to the membrane.

The distances measured by DEER report on the conformation of αS on the membranes. As in the binding studies, the results of the DEER experiments are similar for IMM and NPM, showing that also the conformation of αS is similar on both membranes. As described in the results section, a two-peaked distance distribution fits the data better than a single component, showing that there are two distinct conformations of αS. The long-distance component agrees well with the distance attributed to the extended conformation (Table 2). On IMM and on NPM SUVs, this is the major fraction, which also reveals that more than half of the αS binds to the membrane in the extended conformation. The second fraction has a shorter distance, a distance that is too short for an extended helix conformation. However, the distance is longer than that of the αS horseshoe conformation on micelles (2.7 nm) [23], on SUVs (shown in Fig 2D and Table 2), and on LUVs [30]. The helix 2 appears to be firmly bound, so this fraction cannot be due to a flexible helix 2 section of the protein, and the DLS results show that the SUVs are intact in the presence of αS. Therefore, we attribute this form to a horseshoe-like conformation with a larger opening angle than the horseshoe conformation found on SDS micelles or POPG SUVs and LUVs (Table 2). The molecular interactions leading to this conformation and why it is stabilized by the natural-membrane mimics is difficult to answer. The distance between the helices is too large to enable intramolecular interactions of the sidechains of the helix residues. Specific turn configurations of the residues linking the two helices [48], protein-membrane interactions or the formation of αS-aggregates on the membrane have been discussed as factors leading to the horseshoe conformation, however, so far no conclusive interpretation has been found.

**Table 2:** Parameters of distance distributions for αS27/56 bound to SUVs of IMM and NPM and model membranes for comparison.

SUVs mimicking natural membranes				POPG SUVs		POPG LUVs [30]*	
IMM		NPM					
distance (nm)	fraction (%)	distance (nm)	fraction (%)	distance (nm)	fraction (%)	distance (nm)	fraction (%)
3.7	32	3.6	36	2.6	30	2.7	27
4.2	68	4.3	64	4.2	70	4.3	73

Errors in contribution to fraction ±3% (IMM and NPM) and ±2% (POPG SUVs)

*reanalyzed from ref. (30)

To characterize this novel form in detail, distances between more spin-label pairs would be useful, studies we are planning in the future.

The larger-opening-angle horseshoe conformation is another example of the variability in αS-membrane interaction. The tight binding of αS to the natural membrane-mimics again emphasizes that αS is perfectly suited to interact with such membranes, suggesting that the co-localization and the presumed function may very likely involve these membranes.

## References

[1] Parkinson J. An Essay on the Shaking Palsy (Whitingham and Rowland, London). 1817.

[2] De Rijk MC, Breteler MM, Graveland GA, Ott A, Grobbee DE, van der Meché FG, et al Prevalence of Parkinson’s disease in the elderly: the Rotterdam Study. Neurology. 1995;45: 2143–2146. 8848182 10.1212/wnl.45.12.2143

[3] Junn E, Ronchetti RD, Quezado MM, Kim S-Y, Mouradian MM. Tissue transglutaminase-induced aggregation of alpha-synuclein: Implications for Lewy body formation in Parkinson’s disease and dementia with Lewy bodies. Proc Natl Acad Sci U S A. 2003;100: 2047–2052. 10.1073/pnas.0438021100 12576551 PMC149956

[4] Chung KK, Zhang Y, Lim KL, Tanaka Y, Huang H, Gao J, et al Parkin ubiquitinates the alpha-synuclein-interacting protein, synphilin-1: implications for Lewy-body formation in Parkinson disease. Nat Med. 2001;7: 1144–1150. 10.1038/nm1001-1144 11590439

[5] Spillantini MG, Schmidt ML, Lee VM, Trojanowski JQ, Jakes R, Goedert M. Alpha-synuclein in Lewy bodies. Nature. 1997 pp. 839–840. 10.1038/42166 9278044

[6] Goedert M. Alpha-synuclein and neurodegenerative diseases. Nat Rev Neurosci. 2001;2: 492–501. 10.1038/35081564 11433374

[7] Weinreb PH, Zhen W, Poon AW, Conway K a., Lansbury PT. NACP, a protein implicated in Alzheimer’s disease and learning, is natively unfolded. Biochemistry. 1996;35: 13709–13715. 10.1021/bi961799n 8901511

[8] Uversky VN. Protein folding revisited. A polypeptide chain at the folding—Misfolding—Nonfolding cross-roads: Which way to go? Cell Mol Life Sci. 2003;60: 1852–1871. 10.1007/s00018-003-3096-6 14523548 PMC11146068

[9] Liu S, Ninan I, Antonova I, Battaglia F, Trinchese F, Narasanna A, et al Alpha-Synuclein produces a long-lasting increase in neurotransmitter release. EMBO J. 2004;23: 4506–4516. 10.1038/sj.emboj.7600451 15510220 PMC526467

[10] Maroteaux L, Campanelli JT, Scheller RH. Synuclein: a neuron-specific protein localized to the nucleus and presynaptic nerve terminal. J Neurosci. 1988;8: 2804–2815. 3411354 10.1523/JNEUROSCI.08-08-02804.1988 PMC6569395

[11] Nemani VM, Lu W, Berge V, Nakamura K, Onoa B, Michael K, et al Increased expression of α-Synuclein reduces neurotransmitter release by inhibiting synaptic vesicle reclustering after endocytosis. Neuron. 2010;65: 66–79. 10.1016/j.neuron.2009.12.023 20152114 PMC3119527

[12] Vila M, Ramonet D, Perier C. Mitochondrial alterations in Parkinson’s disease: New clues. J Neurochem. 2008;107: 317–328. 10.1111/j.1471-4159.2008.05604.x 18680555

[13] Devi L, Anandatheerthavarada HK. Mitochondrial trafficking of APP and alpha synuclein: Relevance to mitochondrial dysfunction in Alzheimer’s and Parkinson's diseases. Biochim Biophys Acta. 2010;1802: 11–19. 10.1016/j.bbadis.2009.07.007.Mitochondrial 19619643 PMC2790550

[14] Burré J, Sharma M, Tsetsenis T, Buchman V, Etherton MR, Südhof TC. α -Synuclein promotes SNARE-complex assembly in vivo and in vitro. Science (80-). 2010;329: 1663–1667. 10.1126/science.1195227 20798282 PMC3235365

[15] Kubo SI, Nemani VM, Chalkley RJ, Anthony MD, Hattori N, Mizuno Y, et al A combinatorial code for the interaction of α-synuclein with membranes. J Biol Chem. 2005;280: 31664–31672. 10.1074/jbc.M504894200 16020543

[16] Ardail D, Privat JP, Egret-Charlier M, Levrat C, Lerme F, Louisot P. Mitochondrial contact sites. Lipid composition and dynamics. J Biol Chem. 1990;265: 18797–18802. 2172233

[17] Devi L, Raghavendran V, Prabhu BM, Avadhani NG, Anandatheerthavarada HK. Mitochondrial import and accumulation of alpha-synuclein impair complex I in human dopaminergic neuronal cultures and Parkinson disease brain. J Biol Chem. 2008;283: 9089–9100. 10.1074/jbc.M710012200 18245082 PMC2431021

[18] Nakamura K, Nemani VM, Azarbal F, Skibinski G, Levy JM, Egami K, et al Direct membrane association drives mitochondrial fission by the Parkinson disease-associated protein α-synuclein. J Biol Chem. 2011;286: 20710–20726. 10.1074/jbc.M110.213538 21489994 PMC3121472

[19] Yong-Kee CJ, Sidorova E, Hanif A, Perera G, Nash JE. Mitochondrial dysfunction precedes other sub-cellular abnormalities in an in vitro model linked with cell death in Parkinson’s disease. Neurotox Res. 2012;21: 185–194. 10.1007/s12640-011-9259-6 21773851

[20] Davidson WS, Jonas A, Clayton DF, George JM. Stabilization of alpha-synuclein secondary structure upon binding to synthetic membranes. J Biol Chem. 1998;273: 9443–9449. 10.1074/jbc.273.16.9443 9545270

[21] Eliezer D, Kutluay E, Bussell R, Browne G. Conformational properties of alpha-synuclein in its free and lipid-associated states. J Mol Biol. 2001;307: 1061–1073. 10.1006/jmbi.2001.4538 11286556

[22] Jao CC, Der-Sarkissian A, Chen J, Langen R. Structure of membrane-bound α-synuclein studied by site-directed spin labeling. Proc Natl Acad Sci U S A. 2004;101: 8331–8336. 10.1073/pnas.0400553101 15155902 PMC420394

[23] Ulmer TS, Bax A. structure and dynamics of micelle-bound human alpha-synuclein. J Biol Chem. 2005;280: 9595–9603. 10.1074/jbc.M411805200 15615727

[24] Ulmer TS, Bax A. Comparison of structure and dynamics of micelle-bound human alpha-synuclein and Parkinson disease variants. J Biol Chem. 2005;280: 43179–43187. 10.1074/jbc.M411805200 16166095

[25] Shvadchak V V., Subramaniam V. A four-amino acid linker between repeats in the α-synuclein sequence is important for fibril formation. Biochemistry. 2014;53: 279–281. 10.1021/bi401427t 24397337

[26] Drescher M, Veldhuis G, Van Rooijen BD, Milikisyants S, Subramaniam V, Huber M. Antiparallel arrangement of the helices of vesicle-bound α-synuclein. J Am Chem Soc. 2008;130: 7796–7797. 10.1021/ja801594s 18512917

[27] Lokappa SB, Ulmer TS. Alpha-synuclein populates both elongated and broken helix states on small unilamellar vesicles. J Biol Chem. 2011;286: 21450–21457. 10.1074/jbc.M111.224055 21524999 PMC3122204

[28] Jao CC, Hegde BG, Chen J, Haworth IS, Langen R. Structure of membrane-bound alpha-synuclein from site-directed spin labeling and computational refinement. Proc Natl Acad Sci U S A. 2008;105: 19666–19671. 10.1073/pnas.0807826105 19066219 PMC2605001

[29] Georgieva ER, Ramlall TF, Borbat PP, Freed JH, Eliezer D. Membrane-bound alpha-synuclein forms an extended helix: long-distance pulsed ESR measurements using vesicles, bicelles, and rodlike micelles. J Am Chem Soc. 2008;130: 12856–12857. 10.1021/ja804517m 18774805 PMC2626176

[30] Robotta M, Braun P, Van Rooijen B, Subramaniam V, Huber M, Drescher M. Direct evidence of coexisting horseshoe and extended helix conformations of membrane-bound alpha-Synuclein. ChemPhysChem. 2011;12: 267–269. 10.1002/cphc.201000815 21275016

[31] Robotta M, Hintze C, Schildknecht S, Zijlstra N, Jüngst C, Karreman C, et al Locally resolved membrane binding affinity of the N-terminus of α-synuclein. Biochemistry. 2012;51: 3960–3962. 10.1021/bi300357a 22494024

[32] Middleton ER, Rhoades E. Effects of curvature and composition on α-synuclein binding to lipid vesicles. Biophys J. Biophysical Society; 2010;99: 2279–2288. 10.1016/j.bpj.2010.07.056 20923663 PMC3042580

[33] Rhoades E, Ramlall TF, Webb WW, Eliezer D. Quantification of alpha-synuclein binding to lipid vesicles using fluorescence correlation spectroscopy. Biophys J. Elsevier; 2006;90: 4692–4700. 10.1529/biophysj.105.079251 16581836 PMC1471845

[34] Drescher M, Godschalk F, Veldhuis G, van Rooijen BD, Subramaniam V, Huber M. Spin-label EPR on α-synuclein reveals differences in the membrane binding affinity of the two antiparallel helices. ChemBioChem. 2008;9: 2411–2416. 10.1002/cbic.200800238 18821550

[35] Stefanovic AND, Stöckl MT, Claessens MM a E, Subramaniam V. α-Synuclein oligomers distinctively permeabilize complex model membranes. FEBS J. 2014;281: 2838–2850. 10.1111/febs.12824 24767583

[36] Milov a. D, Tsvetkov YD. Double electron-electron resonance in electron spin echo: Conformations of spin-labeled poly-4-vinilpyridine in glassy solutions. Applied Magnetic Resonance. 1997 pp. 495–504. 10.1007/BF03164129

[37] Van Raaij ME, Segers-Nolten IMJ, Subramaniam V. Quantitative morphological analysis reveals ultrastructural diversity of amyloid fibrils from alpha-synuclein mutants. Biophys J. Biophysical Society; 2006;91: L96–L98. 10.1529/biophysj.106.090449 PMC1635669 16997873

[38] Veldhuis G, Segers-Nolten I, Ferlemann E, Subramaniam V. Single-molecule FRET reveals structural heterogeneity of SDS-bound α-synuclein. ChemBioChem. 2009;10: 436–439. 10.1002/cbic.200800644 19107759

[39] Van Meer G, Voelker DR, Feigenson GW. Membrane lipids: where they are and how they behave. Nat Rev Mol Cell Biol. 2008;9: 112–124. 10.1038/nrm2330 18216768 PMC2642958

[40] Stoll S, Schweiger A. EasySpin, a comprehensive software package for spectral simulation and analysis in EPR. J Magn Reson. 2006;178: 42–55. 10.1016/j.jmr.2005.08.013 16188474

[41] Steigmiller S, Börsch M, Gräber P, Huber M. Distances between the b-subunits in the tether domain of F 0F1-ATP synthase from E. coli. Biochim Biophys Acta—Bioenerg. 2005;1708: 143–153. 10.1016/j.bbabio.2005.03.013 15907787

[42] Jeschke G. Distance measurements in the nanometer range by pulse EPR. ChemPhysChem. 2002;3: 927–932. 10.1002/1439-7641(20021115)3:11<927::AID-CPHC927>3.0.CO;2-Q 12503132

[43] Jeschke G, Chechik V, Ionita P, Godt a., Zimmermann H, Banham J, et al DeerAnalysis2006—a comprehensive software package for analyzing pulsed ELDOR data. Appl Magn Reson. 2006;30: 473–498. 10.1007/BF03166213

[44] Jo E, McLaurin JA, Yip CM, George-Hyslop PS, Fraser PE. α-Synuclein Membrane Interactions and Lipid Specificity. J Biol Chem. 2000;275: 34328–34334. 10.1074/jbc.M004345200 10915790

[45] Zigoneanu IG, Yang YJ, Krois AS, Haque ME, Pielak GJ. Interaction of α-synuclein with vesicles that mimic mitochondrial membranes. Biochim Biophys Acta—Biomembr. Elsevier B.V.; 2012;1818: 512–519. 10.1016/j.bbamem.2011.11.024 PMC3273638 22155643

[46] Robotta M, Gerding HR, Vogel A, Hauser K, Schildknecht S, Karreman C, et al Alpha-Synuclein Binds to the Inner Membrane of Mitochondria in an α-Helical Conformation. Chembiochem. 2014; 1–4. 10.1002/cbic.201402281 25209675

[47] Dikiy I, Eliezer D. N-terminal Acetylation stabilizes N-terminal Helicity in Lipid- and Micelle-bound α-Synuclein and increases its affinity for Physiological Membranes. J Biol Chem. 2014;289: 3652–3665. 10.1074/jbc.M113.512459 24338013 PMC3916564

[48] Bortolus M, Tombolato F, Tessari I, Bisaglia M, Mammi S, Bubacco L, et al Broken helix in vesicle and micelle-bound alpha-synuclein: Insight from site-directed spin labelling-EPR experiments and MD simulations. J Am Chem Soc. 2008;130(21): 6690–91. 10.1021/ja8010429 18457394

